# Early Cessation of Breastfeeding and Determinants: Time to Event Analysis

**DOI:** 10.1155/2020/3819750

**Published:** 2020-04-17

**Authors:** Ebrahim Babaee, Babak Eshrati, Mehran Asadi-Aliabadi, Majid Purabdollah, Marzieh Nojomi

**Affiliations:** ^1^Preventive Medicine and Public Health Research Center, Student Research Committee, Iran University of Medical Sciences, 1449614542 Tehran, Iran; ^2^Preventive Medicine and Public Health Research Center, Department of Community Medicine, Iran University of Medical Sciences, 1449614542 Tehran, Iran; ^3^Department of Nursing, Faculty of Nursing, Khoy University of Medical Sciences, 1449614542 Khoy, Iran

## Abstract

**Background:**

The onset of breastfeeding has a high success rate in most countries, but the time for termination of breastfeeding varies between countries.

**Objective:**

This survey was aimed to determine the effective factors on the early termination of breastfeeding.

**Methods:**

This study was conducted in 2018, in Iran. About 410 mothers were enrolled in the study. All considered factors were evaluated as factors influencing the continuity of breastfeeding. Survival analysis was used to analyze data.

**Results:**

The mean age of the mothers was equal to 29.48 ± 5.8 years. The frequency of termination of breastfeeding before the first 2 years was equal to 34%. The mean of breastfeeding duration was equal to 21.49 ± 5.3 months. The percentage of infants who had been breastfed for 24 months was equal to 65.8%. An infant's birth weight (2500–4000 gr) (hazard ratio: 0.54), neonatal birth order (hazard ratio: 0.69), neonatal pathologic jaundice (hazard ratio: 1.52), starting time of using complementary food (hazard ratio: 2.45), using pacifier (hazard ratio: 2.82), and the status of using artificial milk (hazard ratio: 3.29) were significantly associated with cessation of breastfeeding before 24 months of age. The probability of termination of breastfeeding at 6, 12, 18, and 24 months of age was reported by 6%, 8%, 15%, and 34%, respectively.

**Conclusions:**

There were notifiable variations in breastfeeding rates both in national and international levels. Nevertheless, in this study, the mean of breastfeeding duration was longer compared to a number of countries and previous national studies.

## 1. Background

Breast milk is considered as the first natural food for children, and it has been shown to have nutritional, immunological, and psychological benefits [1]. The Holy Quran emphasizes that mothers should breastfeed their infants for 2 years completely, considering this as the right of the infants [2]. Accordingly, WHO emphasizes that infants should be breastfed for 2 years or more [3].

Breastfeeding is beneficial for the health of both women and newborns [4, 5]. Breastfeeding contributes in increasing the child's intelligence, strengthening the emotional bond between the mother and child, and playing a role in the psychosocial development of the child in the future [6].

The onset of breastfeeding has a high success rate in most countries; for example, in Iran, this is reported to be more than 80%. [7, 8] But, the rate of breastfeeding exclusively decreases over time [9]. Many factors influence the Cessation of Breast Feeding (CBF) in mothers, including the factors related to health care and services [10], diseases affecting the mother and child, multiple births, breastfeeding experience [11], mother's HIV status [11, 12], type of delivery [13], place of birth, birth intervals, and prenatal care [14].

According to the previous studies, early CBF is also associated with spousal support [15], postpartum employment [16], mother's attitude, and knowledge about breastfeeding [17].

Time for termination of breastfeeding is different from one country to another. For example, 60%, 57%, and 12% of mothers stopped breastfeeding before the first 2 years, respectively, in the United States, Iran, and Italy [18–20].

Regarding the breastfeeding program in Iran, implemented since 27 years ago, it should be mentioned that, in Iran, the average of breastfeeding indicators is higher than developing countries, and this is while about 20% of infants under 6 months of age do not have exclusive breastfeeding, and 48% of breastfeeding rate does not continue up to 2 years [8].

Therefore, breastfeeding barriers should be identified and appropriate measures should be taken to resolve them. Hence, this study was designed to determine the effective factors on the early CBF.

## 2. Methods

This study with a registry-based retrospective cohort design was performed in 2018, in Tehran, the capital of Iran, on mothers who breastfed. The Research Council of Iran University of Medical Sciences approved the study. Based on estimated sample size, 410 mothers were enrolled in the study. The subjects were selected by stratified sampling and cluster sampling methods from health centers. Mothers were selected from urban and rural areas and referred to health centers for receiving health care services. All mothers had active electronic health records, all of which were fully documented. A standard checklist was used to extract and collect the information and in each health facility. These records included demographic information and detailed breastfeeding records. All demographic information and effective factors in breastfeeding, as described in details, are presented in Table 1. This information included the age of mothers, infant sex, maternal occupation and education level, type of delivery, duration of breastfeeding, multiple birth status, children's birth order, starting time of using complementary food, status of using artificial milk and using pacifier, and history of neonatal jaundice. All of these variables were evaluated as factors influencing the continuity of breastfeeding. Survival data was related to the mothers who had children under the age of 2 years. The information on termination time of breastfeeding was extracted from the electronic records as an event of interest, and during data collection, the mothers who breastfed were considered as right censored. The interval between the first breastfeeding of the infant by the mother and wean off time was considered as a period for breastfeeding. The duration was calculated in months (from first breastfeeding to 2 years of age) and was considered as the survival time for mothers with the experience of the event of interest. All mothers were informed that their records are being used confidentially for the research. The breastfeeding failure probability was investigated using Life Table, Kaplan–Meier curves, and Log-Rank test. The proportional hazard assumption was tested for all covariates using the Schoenfeld residuals test. Both bivariate and multivariate Cox proportional regression models were used. These models were used to evaluate the relationship between potential risk factors and early CBF. In the survival analysis, the conclusions are made based on the Hazard Ratio (HR), so that if the value is greater than one, and then that group is considered to be at increased risk for occurrence of the event compared to the base group [21]. Data were cleaned, coded, and entered in the software. All statistical analyses were performed at 95% of significance level using Stata software version 14 (Stata Corp, College Station, TX, USA).

## 3. Results

In this survey, a total of 410 mothers who had children less than 2 years of age were included in the study. The mean age of the mothers was equal to 29.48 ± 5.8 years. About 62.4% of mothers were in the age group of 25–35 years. The gender ratio was almost the same in children (female: 49.88%). In this study, most of mothers were housewives with nonacademic literacy, and only 8.4% of them were illiterate (Table 1).

**Figure 1:**
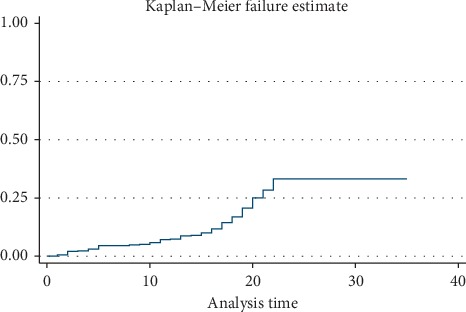
K–M failure estimate of breastfeeding.

The cumulative failure probability presented in Life Table indicated the percentage of 34% for children with breastfeeding failure before the first 2 years (95% CI: 0.29, 0.39). The ratio of breastfeeding immediately after birth and during the first month was reported by 99% (95% CI: 0.97, 0.99). Additional information is presented in Table 2. The mean of breastfeeding duration was equal to 21.49 ± 5.3 months. The duration of breastfeeding in 34.2% of infants was less than 24 months. In this survey, 14.8% and 17.8% of infants were reported to have the history of using artificial milk and pacifier, respectively. Also, the starting time of using complementary food before 6 months of age was reported by 9.5% (Table 1). The Kaplan–Meier failure probability curve is presented in Figure 1.

In the Cox proportional regression model, infant sex, infants̓ birth weight, neonatal birth order, neonatal pathogenic jaundice, starting time of using complementary food, using pacifier, and status of using artificial milk were considered as the independent predictors of CBF before 2 years of age (*p* < 2). The results of the effect of these prognostic factors on the breastfeeding failure probability obtained based on the Cox proportional hazard model are presented in Table 3. According to this approach, infants̓ birth weight (2500–4000 gr) (hazard ratio (HR), 0.54), neonatal birth order (hazard ratio (HR), 0.69), neonatal pathologic jaundice (hazard ratio (HR), 1.52), starting time of using complementary food (hazard ratio (HR), 2.45), using pacifier (hazard ratio (HR), 2.82), and status of using artificial milk (hazard ratio (HR), 3.29) were significantly associated with CBF before 24 months of age. The proportional hazard assumption was tested using the Schoenfeld residuals test. The proportional hazard assumption was justified, since the test showed no significance level (*P* > 0.05) for all covariates. Estimated Kaplan–Meier failure probability of breastfeeding is shown in Figure 1. The adjusted results showed that neonatal pathologic jaundice (hazard ratio (HR), 1.87; 95% CI: 1.22–2.85), starting time of using complementary food (hazard ratio (HR), 1.89; 95% CI: 1.08–3.31), and status of using artificial milk (hazard ratio (HR), 3.43; 95% CI: 1.02–11.57) were significantly associated with CBF (Table 3). The results of the survival analysis showed that the probability of termination of breastfeeding at 6, 12, 18, and 24 months of age was equal to 6%, 8%, 15%, and 34%, respectively (Table 2).

## 4. Discussion

Despite the important activities of Iranian authorities conducted to promote the health status of mothers and children, there are notifiable variations in lactation rates, which are not easily justifiable [22]. In this survey, 395 mother-child pairs were followed retrospectively for a total of 8110 person-months. The incidence rate of CBF before 2 years of age was reported 16.02 person-months. This was somewhat higher than that found in Ethiopia in 2016. In the study conducted in Ethiopia, the incidence rate was reported 13.70 person-months [23]. The findings of the present study showed that one-third of the mothers (34.2%) ceased breastfeeding before 24 months of age and the probability of termination of breastfeeding at 24 months of age was equal to 34%. The results of a study showed that, in Iran and Australia, mothers discontinue lactating earlier than recommended time. [2]. In a study carried out in 2012 at a national level, a mean of 57% of infants had been breastfed for 24 months [22]. Similar to the present study, this study was also conducted among urban and rural residents. In this regard, sociodemographic differences can be particularly effective. Compared to this, in the current study, the mean of infants who had been breastfed for 24 months was reported as 65.8%. The rate of early discontinuation of breastfeeding (before 6 months of age) in the present study (6%) was similar to the that of previous national studies in 2012 [19]. In this study, the risk of early CBF was found to be lower in male infants.This issue cannot be clinically justified, and also the estimated effect size was not statistically significant. In this study, with the increase in the age of mothers, the probability of lactation increased. It can be due to the high level of lactation experience in older women. This positive correlation between maternal age and duration of breastfeeding has been shown in several studies [7, 24], although this was not observed in the study by Olang et al. [19]. The present study demonstrated that the housewife-mothers had stopped breastfeeding earlier compared to the employed mothers. This finding is not in agreement with the results of the studies conducted in Greece, Ethiopia, and Australia. As these studies suggested that postpartum employment within a short time (3 months), in addition to the lack of breastfeeding rooms in working places caused the early CBF in case of mothers who were government employees [23–25]. Considering this, it should be noted that the results of the present study, due to the small sample size in the employed mothers group (*N* = 10), cannot be valid. In line with the results of a study implemented in India [26], the results of the current study showed that duration of breastfeeding was less in better-educated mothers than low-educated mothers. This increase in lactation was nearly double in illiterate women than in women with college education. This finding coincided with the results of other Iranian studies [27]. Relative to the other studies, findings of the surveys conducted in South Africa and Kuwait showed that the breastfeeding duration increases with the increase in the educational level of mothers [11, 28]. This difference can be attributed to the early starting time of complementary feeding among better-educated mothers leading to early CBF [23]. The findings of the present study demonstrated that high infant birth weight is a predictor of increased breastfeeding in the coming months, although the results were not statistically significant. The findings of the study revealed that the probability of lactation continuity for the first child was about half of the probability for the second and more children. Similar to the factor of age of the mother, this increase can be due to the high level of lactation experience of mothers in second and more children. The results of some studies carried in Ethiopia and Bangladesh showed that the breastfeeding duration was higher in mothers with 4 children than the mothers with more than 4 children. It has been claimed that having a small number of children results in having enough time for breastfeeding [13, 23]. It was found that the duration of breastfeeding in women with vaginal delivery was approximately the same as in cesarean section. But, some studies have confirmed that delivery by cesarean section is a risk factor for early CBF [29]. In this case, some studies also showed similar results to the present study [30]. In the following, neonatal pathologic jaundice is introduced as a factor found to be significantly related to early breastfeeding cessation. After an extensive review, no study was found which has evaluated this issue. Despite the notifiable benefits of breastfeeding, several valid studies have demonstrated that the risk of increasing the neonatal hyperbilirubinemia is strongly associated with breastfeeding [31]. It is recommended to conduct more complete studies to evaluate the effect of pathologic jaundice on the continuation of breastfeeding. The use of pacifiers has been shown to have a negative effect on breastfeeding duration [24], which was also confirmed in the present study like a previous internal study conducted in 2012 [19]. It should be noted that a number of studies claimed that the effect of pacifier exposure on the duration of any breastfeeding is a debated topic. In case of breastfeeding infants, the WHO strongly recommends that the pacifier should not be used at all. [32]. The results of the study showed that using pacifier and artificial milk significantly increased the probability of early CBF before 2 years of age. Possibly, the infants̓ status of using artificial milk can be attributed to the inadequacy of breast milk. Finally, it is noteworthy that, in this study, compared to other studies, relatively different factors were studied to assess their effect on lactation. Nevertheless, further studies are needed to obtain more detailed surveys in this regard.

Unlike many studies, this study evaluated the probability of lactation failure at different times up to 2 years of age. Also, the studied predictive factors were relatively different from the factors assessed in previous similar studies. Our study had some limitations and potential biases, which are as follows: recall bias was considered as a limitation in the current study, which might lead to overestimation or underestimation of the results. Another limitation was that the participants were recruited only from a single geographic location, which might cause the results not to be generalizable at the national level. Despite the limitations mentioned above, the present study is valuable because it was carried out among people with middle level of income, which may yield limited evidence, but it is clear that these types of studies can better reveal the prevalence and predictive factors in relation to breastfeeding, viewed as an important issue in the field of health care.

## 5. Conclusions

The lactation situation seems to be relatively good in Iran compared to several other countries [22]. But, according to the results of several surveys and the findings of this study, it appears that the situation of breastfeeding is relatively far from WHO recommendations in many countries as well as in Iran. Nevertheless, in this study, the mean duration of breastfeeding was longer compared to a number of countries and the previous national studies. Therefore, educational empowerment of mothers about the mentioned determinants of breastfeeding can be effectively useful to increase breastfeeding duration.

## Figures and Tables

**Table 1:** Characteristics of the neonates and mothers with a history of breastfeeding (*n* = 410).

Variables	Number^a^	
Infant sex		
Male	203	(49.9)
Female	204	(50.1)

Maternal age group (yr)		
<25	89	(22.2)
25–35	251	(62.4)
>35	62	(15.4)

Maternal employment		
Employed	10	(2.5)
Housekeeper	397	(97.5)

Maternal education		
Academic	145	(35.8)
Nonacademic literate	226	(55.8)
Illiterate	34	(8.4)

Infant birth weight (gr)		
<2500	22	(5.5)
2500–4000	364	(91.5)
>4000	12	(3)

Neonatal birth order		
1st	204	(50.4)
≥2st	201	(49.6)

Delivery type		
Vaginal	204	(50.4)
Cesarean section	201	(49.6)

Neonatal pathologic jaundice		
No	319	(78.4)
Yes	88	(21.6)

Artificial milk using		
No	346	(85.2)
Yes	60	(14.8)

Complementary food starting		
≥6 months	364	(90.5)
<6 months	38	(9.5)

Pacifier using		
No	337	(82.2)
Yes	73	(17.8)

Breastfeeding duration		
<24 months	138	(34.2)
≥24 months	266	(65.8)

^a^The sum of subgroup may be less than total due to missing data.

**Table 2:** Failure probability of breastfeeding (overall).

Time intervals (month)	Population	Event	Lost	Cum. Failure	95% CI
6	379	6	0	0.06	(0.04–0.08)
12	368	5	0	0.08	(0.06–0.11)
18	345	10	0	0.15	(0.12–0.19)
24	261	0	255	0.34	(0.29–0.39)

**Table 3:** Effect of various predictive factors on failure probability of breastfeeding using the univariate (unadjusted) and multivariate (adjusted) Cox regression model.

Variables	Unadjusted HR (95% CI)	*P* value	Adjusted HR (95% CI)^a^	*P* value
*Infant sex*				
Female	1.00		1.00	
Male	0.74 (0.52–1.04)	0.08	0.72 (0.49–1.05)	0.09

*Maternal age group* (yr)				
<25	1.00		1.00	
25–35	0.93 (0.62–1.42)	0.75	1.17 (0.72–1.92)	0.51
>35	0.71 (0.38–1.31)	0.27	0.72 (0.35–1.47)	0.36

*Maternal employment*				
Employed	1.00		1.00	
Housekeeper	1.7 (0.42–6.87)	0.45	3.42 (0.79–14.83)	0.09

*Maternal education*				
Academic	1.00		1.00	
Nonacademic literate	1.14 (0.79–1.64)	0.48	1.45 (0.96–2.21)	0.07
Illiterate	0.54 (0.23–1.27)	0.16	0.79 (0.31–2.01)	0.62

*Infant birth weight* (gr)				
<2500	1.00		1.00	
2500–4000	0.54 (0.29–0.99)	0.04	1.29 (0.58–2.85)	0.53
>4000	0.23 (0.05–1.05)	0.05	0.69 (0.31–3.48)	0.66

*Neonatal birth order*				
1st	1.00		1.00	
≥2st	0.69 (0.48–0.98)	0.03	0.82 (0.53–1.27)	0.38

*Delivery Type*				

Vaginal	1.00		1.00	
Cesarean section	1.11 (0.78–1.57)	0.56	0.98 (0.66–1.44)	0.91
*Neonatal pathologic jaundice*				
No	1.00		1.00	
Yes	1.52 (1.04–2.23)	0.03	1.87 (1.22–2.85)	0.003

*Complementary food starting*				
≥ 6 months	1.00		1.00	
<6 months	2.45 (1.53–3.09)	<0.001	1.89 (1.08–3.31)	0.02

*Pacifier using*				
No	1.00		1.00	
Yes	2.82 (1.92–4.13)	<0.001	0.93 (0.29–2.91)	0.89

*Artificial milk using*				
No	1.00		1.00	
Yes	3.29 (2.22–4.89)	<0.001	3.43 (1.02–11.57)	0.04

^a^Adjusted for all variables in the table.

## Data Availability

Data can be obtained from the corresponding author upon request.
